# The potential of integrative phenomics to harness underutilized crops for improving stress resilience

**DOI:** 10.3389/fpls.2023.1216337

**Published:** 2023-06-20

**Authors:** Dominik K. Großkinsky, Jean-Denis Faure, Yves Gibon, Richard P. Haslam, Björn Usadel, Federica Zanetti, Claudia Jonak

**Affiliations:** ^1^ AIT Austrian Institute of Technology, Center for Health and Bioresources, Bioresources Unit, Tulln a. d. Donau, Austria; ^2^ Université Paris-Saclay, INRAE, AgroParisTech, Institut Jean-Pierre Bourgin, Versailles, France; ^3^ INRAE, Univ. Bordeaux, UMR BFP, Villenave d’Ornon, France; ^4^ Bordeaux Metabolome, INRAE, Univ. Bordeaux, Villenave d’Ornon, France; ^5^ Rothamsted Research, Harpenden, Hertfordshire, United Kingdom; ^6^ IBG-4 Bioinformatics, CEPLAS, Forschungszentrum, Jülich, Germany; ^7^ Biological Data Science, Heinrich Heine University, Universitätsstrasse 1, Düsseldorf, Germany; ^8^ Department of Agricultural and Food Sciences (DISTAL), Alma Mater Studiorum - Università di Bologna, Bologna, Italy

**Keywords:** agriculture, breeding, climate change resilience, crop improvement, neglected crops, phenomics, sustainability

## 1 Introduction

The current agricultural and food system faces diverse and increasing challenges. These include feeding an ever-growing human population, expected to reach about 10 billion by 2050 combined with societal disruption, and the need to cope with the impact of climate change (FAO, 2022). Given that future environmental conditions will limit crop productivity (Zhao et al., 2017; Cooper et al., 2021) and the limited potential to continually increase the performance of staple crops by conventional breeding (Hickey et al., 2019), there is an urgent need to transform agricultural systems. Central to this transformation is the application of alternative, accelerated, and sustainable approaches for the improvement and development of underutilized crops (Hickey et al., 2019). Modern breeding strategies for major crops have widely integrated novel technologies, such as advanced phenotyping or genome-wide interactions, and even epigenomics within “beyond the gene” strategies (Crisp et al., 2022) to speed up crop/genotype selection (Hickey et al., 2019; Kumar et al., 2023). Deploying phenotyping at different scales has the potential to identify novel trait(s) components that can be targeted to accelerate crop improvement (Araus and Cairns, 2014; Großkinsky et al., 2015b; Zhao et al., 2019; Varshney et al., 2021). There is even greater potential for these technologies when used to improve underutilized crops and support the agricultural transformation, as underutilized crops typically lack a biased breeding/selection history, i.e., they often exhibit a high genetic diversity and potential, and are usually better adapted to challenging environments (Kumar et al., 2021; Kumar et al., 2023). To illustrate the application of an integrative phenomics approach we discuss how combining multi-omics and advanced phenotyping is being applied to the underutilized oilseed crop *Camelina sativa* (camelina, gold-of-pleasure, false flax) to facilitate the generation of climate-smart crops for future agricultural systems.

## 2 Novel technologies and approaches for crop improvement

The time needed to select new varieties or species, but also to set up new agronomic practices (e.g., reduced input management) is currently too long in view of the rapidly changing climate^1^. While traditionally varieties have often been selected in a context of high input mechanized monoculture, it is now necessary to develop predictive strategies to formulate the genotypes and agronomic practices adapted to low input, sustainable and diversified cultures. That said, plant biologists have long been studying adaptation or acclimation to stresses associated with climate change (e.g., drought, heat, attacks from pests and pathogens) to unravel the involved mechanisms and identify targets to improve crop performance.

*Reverse genetics* usually starts from a gene and characterizes associated phenotypes. Here, the development of “omics” has revolutionized biology over the past 20 years and led to a dramatic increase in the number of candidate genes and networks (e.g., Kajala et al., 2021). Great progress was initially achieved in Arabidopsis but has increasingly been translated to major crops (Fernie and Tohge, 2017). Bottom-up systems biology goes one step further by assembling reductionist knowledge using models to explain phenotypes (Keurentjes et al., 2011). One consequence of this is that more complex mechanisms can be analysed, and more sophisticated improvement strategies proposed (Chen et al., 2021). This ensemble of approaches is being carried out in a growing number of species (Stevens et al., 2018; Hawkins et al., 2021). However, when going beyond model plants and major established crops, challenges remain. Whilst resolving homozygous genomes (van Rengs et al., 2021) and pangenomes (Bayer et al., 2020) of crops with a medium genome size is now achievable with the advent of novel sequencing technologies (Mascher et al., 2021), challenges remain with consistent gene annotation and ontology developments. Rapid progress is being made with more mature tools, facilitating structural (Holst et al., 2023) and functional genome annotation (Bolger et al., 2018; Schwacke et al., 2019), whilst plant genome data continues to be gathered in comprehensive databases (Vandepoele, 2017). However, several problems remain outstanding, including the accurate description of phenotypes together with necessary metadata (Papoutsoglou et al., 2020) and simple data management approaches (Pommier et al., 2019; Jacob et al., 2020). This is especially true for minor or even orphan crops where, without the necessary resources, these strategies have not yet reached the same level of maturity as for major field crops (Watt et al., 2020). To address this, remote sensing, deep phenotyping, and machine learning approaches are continually being developed for crops (Fernie et al., 2021; Wong et al., 2023).

*Forward genetics* starts from the phenotype and tries to identify the underlying mechanisms, genes and/or markers. Traditionally, crop improvement has been based on traits of interest, especially yield. The most promising lines are retained, which results in an enrichment of the germplasm with performance-promoting alleles. More recently, proxies such as plant height have started to be used to speed up the process (Madec et al., 2017). The aim being to find phenotype-based parameters that are easy and cheap to measure. Classical selection in the field using plots is very expensive and generally subject to seasons and environmental hazards. It is in this context that multiple phenotyping facilities have been established^2^. By enabling plant phenotyping under controlled conditions, their purpose is to generate field performance proxies at affordable cost (Reynolds et al., 2019), although the confirmation of agronomic trait evaluations carried out under controlled conditions in the field is challenging (Hohmann et al., 2016). Indeed, when transferring phenotypic information from controlled environments to agronomic field conditions, phenotypes are not always confirmed (Shi et al., 2017; Zhang et al., 2019; Metje-Sprink et al., 2020). For example, evaluations of edited plants that have targeted mutations for specific and well-characterized traits have shown limitations, as only 13 out of 22 publications confirmed yield effects of edited plants identified in controlled environments in subsequent field trials (Metje-Sprink et al., 2020). Therefore, field trials remain essential for the study of traits interacting with complex environments. Phenotyping under controlled conditions requires continued evaluation of growth scenarios to determine the best predictive variables. Over the past decade more and more sophisticated sensors and platforms have emerged. Whether in fields or greenhouses, they use an ever-increasing number of imaging techniques (Großkinsky et al., 2015a; Brichet et al., 2017; Yang et al., 2020; Dodig et al., 2021) as non-destructive tools that allow the generation of performance proxies throughout plant growth (e.g., Millet et al., 2019). Genomic selection (e.g., R2D2 Consortium et al., 2021), increasingly used by breeders, consists of predicting the genetic value of candidates for selection through the development of models linking phenotype and genotype. The advantage of this approach is that the phenotyping step can be done with a small subset of the germplasm and the search for potentially interesting genotypes (pre-breeding) can be done with young plants and therefore at low cost. Paradoxically, sampling of plant material is still largely manual and the development of robots for destructive-type observations is still in its infancy (Foix et al., 2018). This may be one of the reasons why “omics” are not yet fully considered as selection tools, in particular metabolomics, whose costs have fallen sharply (López-Ruiz et al., 2019). Evidence continues to emerge demonstrating that transcriptomics or metabolomics (Westhues et al., 2017) can carry more condensed information than the genome, and these approaches can be used to build top-down models capable of predicting traits, particularly in integrative approaches combining omics technologies with advanced phenotyping.

## 3 Underutilized crops and their agricultural potential

Despite a huge variety of cultivated plants, historical developments in global agriculture have resulted in a very narrow diversity of crops contributing to our current food system, with only few species accounting for over 90% of world food production, e.g., the staple cereals rice, wheat, and maize (Milla and Osborne, 2021). In contrast, the vast majority of more than 5000 known edible crops are only cultivated on a small scale, if at all, and are largely neglected in modern agricultural settings due to their limited competitiveness (Padulosi, 2017). These wild, cultivated, or semi-domesticated crops are not yet fully integrated into typical agricultural systems worldwide (Padulosi, 2017). These crops are typically underutilized in agriculture due to a lack of improvement and in-depth agronomic knowledge and/or experience, moreover, they are poorly represented in fundamental research, which is mostly focused on model plants or staple crops. Thus, there is often a lack of complementary knowledge, technologies, and resources from basic research to support use and integration of such crops. Underutilised crops (for examples see **Table 1**) have recently gained attention as many of them hold untapped potential for coping with the challenges of current and future agriculture (Padulosi, 2017; Kumar et al., 2023), e.g., increasing agro-biodiversity, improving the overall resilience of agricultural systems against climate change and sourcing alternative (high-quality) food ingredients. This is evident in research today e.g., EU-funded projects, aiming to promote the use of underutilized crops, such as INCREASE^3^ aiming at improving the sustainable use of the pulses chickpea, common bean, lentil and lupin, PROTEIN2FOOD^4^ addressing the development and sustainable use of quinoa, amaranth, buckwheat and pulses like lupins, and UNTWIST^5^ aiming at the promotion of the sustainable use of camelina as a promising climate-resilient oilseed crop. All these projects focus on crop phenotypes and environmental plasticity.

**Table 1:** Examples of promising underutilized crops exploitable for agricultural use.

Underutilized crop	Origin	Alternative to	Purpose / Advantage	Suitable for
Amaranth	Global	Wheat/cereals	Alternative grain, protein source, gluten-free	Dry, hot tropical to temperate climates
Buckwheat	Asia	Cereals	Alternative grain, protein source, gluten-free	Cool, moist climates
Camelina	Europe	Rapeseed, soybean	Alternative oilseed, high oil quality, protein source, climate-resilient	Marginal land/poor soils, dry, warm to cold, not too moist climates
Carinata	Africa	Rapeseed, soybean	Alternative oilseed, aviation/biofuel production	Marginal land, dry climates
Cowpea	Africa, Asia	Other legumes	Alternative protein	Dry climates, sandy soils
Cucurbits	Americas	Oilseeds, (sweet) potato	High-quality seed oil, nutritional value of flesh	Warm, dry, not too moist climates
Linseed	Global	Oilseeds, cotton	High-quality oil, natural fibres	Wet, not too warm climates
Lupin	Africa, Andes, Europe	Other legumes	Alternative protein	Dry, not too warm climates, high sun exposure, acidic soils (some types)
Millets	Global	Cereals	High nutritional value, climate-resilient, gluten-free	Dry, hot climates, areas of high flood risk
Pennycress	Americas, Eurasia	Oilseeds	Alternative oilseed, protein source	Wet, cold climates
Quinoa	Andes	Wheat/cereals	Alternative grain, protein source, gluten-free	Dry, not too warm climates, mountainous regions, saline soils
Sorghum	Global	Cereals	Alternative grain, gluten-free	Hot, dry climates, saline soils
Teff	Africa	Wheat/cereals	Alternative grain, protein source, gluten-free	Dry, hot climates, poor soil, areas of high waterlogging risk

Among underutilized crops with significant potential, camelina has recently attracted interest as a promising alternative to other oilseeds (e.g., rapeseed). From a scientific perspective, this is also due to its genetic similarity with the model plant Arabidopsis (Kagale et al., 2014). Camelina can be easily integrated into existing agricultural systems (Zanetti et al., 2021). It has a natural capacity to withstand harsh environmental conditions, and tolerance to pests and diseases represent additional features that make it a suitable crop for many environments worldwide (Berti et al., 2016; Zanetti et al., 2021), except for too wet conditions (Stasnik et al., 2022). This particularity also makes it a potential source of resistance mechanisms that would have disappeared or have become insufficient in species that have been selected above all for their yield in optimal conditions. So, the aim here is not to rediscover mechanisms that are already known. The emerging interest in camelina has spurred genomic and genetic investigations, including the description of a reference genome (Kagale et al., 2014). The development of Recombinant Inbred Lines (RILs) has further allowed the identification of important QTLs e.g., 1000-seed weight, seed number per pod, days to flowering or seed oil content (King et al., 2019; Li et al., 2021). Nevertheless, the throughput of these approaches is limited particularly at the phenotype level, as for other (underutilized) crops. Hence, further focused improvement of camelina is required and the use of novel approaches and technologies, specifically integrating the crop phenotype, is necessary and achievable as respective protocols for high-throughput phenotyping are established (Vello et al., 2022).

## 4 Integrative phenomics to unlock the potential of the oilseed crop camelina as a blueprint to improve stress tolerance

Several projects are emerging that take advantage of the availability and affordability of modern technologies (e.g., multi-omics, phenomics, network discovery) to investigate plant plasticity in novel crops (e.g., Girija et al., 2021; Marks et al., 2021). Understanding plant plasticity is key for the development of novel crops for resilient food production systems in increasingly variable climates. As an exemplary project, UNTWIST^5^ targets camelina to develop a systems-based understanding of the key mechanisms underlying crop stress tolerance using a phenomics approach. The project is based on the premise that the unravelling of stress adaptation mechanisms in naturally resilient camelina (Zanetti et al., 2021), which has not yet undergone intensive breeding, will reveal successful stress adaptation strategies. Research is centred on agronomically relevant stress scenarios (linking experiments in controlled environments and field conditions) to improve our understanding of plant responses to challenging growth conditions. Therefore, diverse germplasm is analysed at the genomic, epigenetic, transcriptional, physiological, proteomic, and metabolomic level (incl. lipidome, redox status) complemented by agronomic parameters and advanced non-invasive multi-sensor phenotyping. Despite limited basic knowledge on camelina compared to well-studied plant species, this integrative multi-omics approach deploying phenotyping from cell to whole plant level (Dhont et al., 2013; Ghanem et al., 2015; Großkinsky et al., 2015b) allows the identification and dissection of mechanisms contributing to camelina performance under challenging environments representative of future conditions. Integration of the multi-layered data serves modelling approaches and supports potential marker development for crop improvement under adverse climate conditions. Furthermore, the results will underpin databases, protocols, and advice for downstream application. The new knowledge can be utilized for further improvements of camelina and other (underutilized) crops in combination with modern strategies in breeding and engineering crops (Jiang et al., 2017; Morineau et al., 2017; Lemmon et al., 2018; Fernie and Yan, 2020; Rönspies et al., 2021; Yu et al., 2021; Bellec et al., 2022; Han et al., 2022).

## 5 Discussion

Global agriculture urgently requires alternative strategies in crop development and improvement. So far, the great potential of underutilized crops to contribute to the agricultural transition in our food systems remains untapped. This is due to a lack of knowledge and appropriate technologies; shortfalls that are now being addressed. Recognizing that phenotypes are as or even more important than genotypic information has driven recent advances in crop phenotyping. These advances greatly contribute to the development of multi-omics systems-based phenomics approaches, which have shown their potential for accessing previously untapped resources and can be applied more directly to underutilized non-staple crops in the future, significantly reducing the dependency on information from staple crops and model plants as the only information source. This allows the effective improvement and utilization of such crops to support the re-configuration of agricultural and food systems in a sustainable manner. Most notably, phenomics and multi-omics approaches will enable agriculture to harness the potential of underutilized crops. Projects like UNTWIST, which apply these approaches, are needed to prove, and validate the potential of these novel tools. Ultimately, such projects can serve as a blueprint for improving underutilized crops and provide a platform for establishing more climate-resilient food production systems.

## Author contributions

All authors jointly defined the outline of the manuscript. DKG drafted the manuscript with input from all authors. All authors contributed to the article and approved the submitted version.

## Footnotes

1
https://climate.copernicus.eu/esotc/2022

2
www.plant-phenotyping.org

3
www.pulsesincrease.eu

4
www.protein2food.eu

5
www.untwist.eu
